# Differences between physician and patient preferences for cancer treatments: a systematic review

**DOI:** 10.1186/s12885-023-11598-4

**Published:** 2023-11-18

**Authors:** Mengqian Zhang, Xiaoning He, Jing Wu, Feng Xie

**Affiliations:** 1 School of Pharmaceutical Science and Technology, Tianjin University, No 92 Weijin Road, Nankai District, Tianjin, CO 300072 China; 2 Center for Social Science Survey and Data, Tianjin University, Tianjin, China; 3 Department of Health Research Methods, Evidence and Impact, McMaster University, Hamilton, ON Canada; 4 Centre for Health Economics and Policy Analysis, McMaster University, Hamilton, ON Canada

**Keywords:** Cancer treatment decision-making, Preference difference, Systematic review

## Abstract

**Background:**

Shared decision-making is useful to facilitate cancer treatment decisions. However, it is difficult to make treatment decisions when physician and patient preferences are different. This review aimed to summarize and compare the preferences for cancer treatments between physicians and patients.

**Methods:**

A systematic literature search was conducted on PubMed, Embase, PsycINFO, CINAHL and Scopus. Studies elicited and compared preferences for cancer treatments between physicians and patients were included. Information about the study design and preference measuring attributes or questions were extracted. The available relative rank of every attribute in discrete choice experiment (DCE) studies and answers to preference measuring questions in non-DCE studies were summarized followed by a narrative synthesis to reflect the preference differences.

**Results:**

Of 12,959 studies identified, 8290 were included in the title and abstract screening and 48 were included in the full text screening. Included 37 studies measured the preferences from six treatment-related aspects: health benefit, adverse effects, treatment process, cost, impact on quality of life, and provider qualification. The trade-off between health benefit and adverse effects was the main focus of the included studies. DCE studies showed patients gave a higher rank on health benefit and treatment process, while physicians gave a higher rank on adverse effects. Non-DCE studies suggested that patients were willing to take a higher risk of adverse effects or lower health benefit than physicians when accepting a treatment.

**Conclusions:**

Physicians and patients had important preference differences for cancer treatment. More sufficient communication is needed in cancer treatment decision-making.

**Supplementary Information:**

The online version contains supplementary material available at 10.1186/s12885-023-11598-4.

## Background

Cancer patients often need to choose from multiple treatment options with various health benefit and safety profiles. Patient preference thus plays an important role in such decision making [1]. Shared decision-making (SDM) explicitly considers patient preference and value and has been increasingly used in cancer care practice [2–4]. SDM involves the interaction and mutual information sharing between physicians and patients, where physicians provide evidence-based and rational treatment messages, and patients express their needs and preferences [5]. Through SDM, all useful information is considered and treatments are selected based on preferences, which helps to improve the treatment compliance and outcome [6, 7]. However, this decision-making process becomes difficult when physician and patient’s preferences differ.

A few reviews have investigated how patients’ preferences were different from those of physicians. Montgomery and Fahey suggested that discordant preferences between patients and physicians always existed, and the magnitude of the differences varied with disease conditions [8]. Muhlbach and Juhnke also reported mixed degrees of differences between the preferences of patients and the judgements of physicians, where the physician judgements were defined as their evaluation on patients which is different from physician preferences [9]. Harrison et al. reviewed studies using discrete choice experiment (DCE) to elicit both patient and healthcare provider preferences, and found that healthcare providers weighed more on treatment outcome (e.g., mortality) and treatment structure (e.g., organizational structures, human resources), while patients placed more weights on the treatment process (e.g., risk, treatment regimen, waiting time) [10].

However, there lacks a comprehensive comparison of treatment preferences between physicians and patients with cancer. Therefore, we conducted a systematic literature review aimed at comparing patient preferences for cancer treatment with those of physicians.

## Methods

This review was structured in accordance with the Preferred Reporting Items for Systematic Reviews and Meta-Analyses (PRISMA) guidelines [11].

### Search strategy

A systematic literature search was conducted on PubMed, EMBASE, PsycINFO, CINAHL and Scopus, from the inception of the databases to May 4, 2022. The search strategy combined Medical Subject Headings (MeSH) terms about “neoplasm” and free text pertaining to “cancer”, “physician”, “patient” and “preference”. Further search details can be found in Appendix 1. Reference lists of included papers were also manually searched.

### Inclusion and exclusion criteria

Studies were included if they directly compared physician and patient preferences for cancer treatments using established methods, including DCE, conjoint analysis (CA), the threshold technique, time trade-off (TTO), trade-off method (TTM), standard gamble (SG), prospective measure of preference (PMP), and self-designed questionnaire. Patients were those who were having cancer and facing treatment decisions.

Studies were excluded if they measured preferences for other health conditions (e.g., cancer-related chronic pains); measured preferences for cancer screening or diagnosis, instead of cancer treatments; or elicited preference from proxies (e.g., general public or family members). Non-research articles, including conference abstracts, letters, and editorials, as well as reviews were also excluded.

### Study selection

Both title and abstract and full text screenings were conducted independently and in duplicate by two reviewers (MZ and XH). Any discrepancies between reviewers were discussed and resolved through consensus. If necessary, a third reviewer (JW) was consulted to make the final decision.

### Appraisal and quality assessment

The International Society for Pharmacoeconomics and Outcomes Research (ISPOR) Good Research Practices for Conjoint Analysis Task Force was used to assess the DCE and CA studies, and the Appraisal Tool for Cross-Sectional Studies (AXIS) was used to assess other studies (Appendix 2 and 3) [12, 13]. Two reviewers (MZ and XN) independently applied the guide/tool to each included study and recorded supporting information and justifications for assessments. Any discrepancies in judgements were resolved through consensus, with a third reviewer (JW) acting as an arbiter if necessary.

### Data extraction and synthesis

A narrative synthesis of the included studies was conducted given the heterogeneity among these studies [14]. Basic information of each included study was extracted, including first author, publication year, study country, cancer type, elicitation technique, sample recruitment approach, sample size and mode of administration.

In DCE/CA studies, attributes and their levels were pre-defined, describing the alternative scenarios for participants, to investigate preferences, while in non-DCE/non-CA studies, generic or trade-off questions were used. These attributes and questions were abstracted and grouped into 6 categories in line with the systems-based framework which was used to assess the quality of healthcare [15] and operationalized in previous reviews in this area [9, 16, 17]: (1) health benefits –patients’ health outcomes and clinical benefits; (2) adverse effects – mainly treatment induced side effects; (3) treatment process – process-related factors (e.g., dosage form, dosing frequency, etc.); (4) cost – any types of treatment costs; (5) impact on quality of life - influences developed by treatment on patients’ daily activities and physical or psychological conditions; and (6) provider qualification – type of healthcare organization (e.g., specialist hospital, general hospital, etc.) and reputation of medical personnel.

Differences in preferences for cancer treatment between physicians and patients were summarized. For DCE/CA studies, the ranking of attributes, if reported, was extracted. For threshold technique/TTO/TTM studies, the threshold scores were extracted. For SG/PMP studies, the willingness-to-trade values were extracted. For questionnaire studies, the proportions of participants to specific question options were exacted. If statistic test was conducted to verify the significant difference between physicians and patients, corresponding P-values were extracted.

## Results

**Fig. 1:**
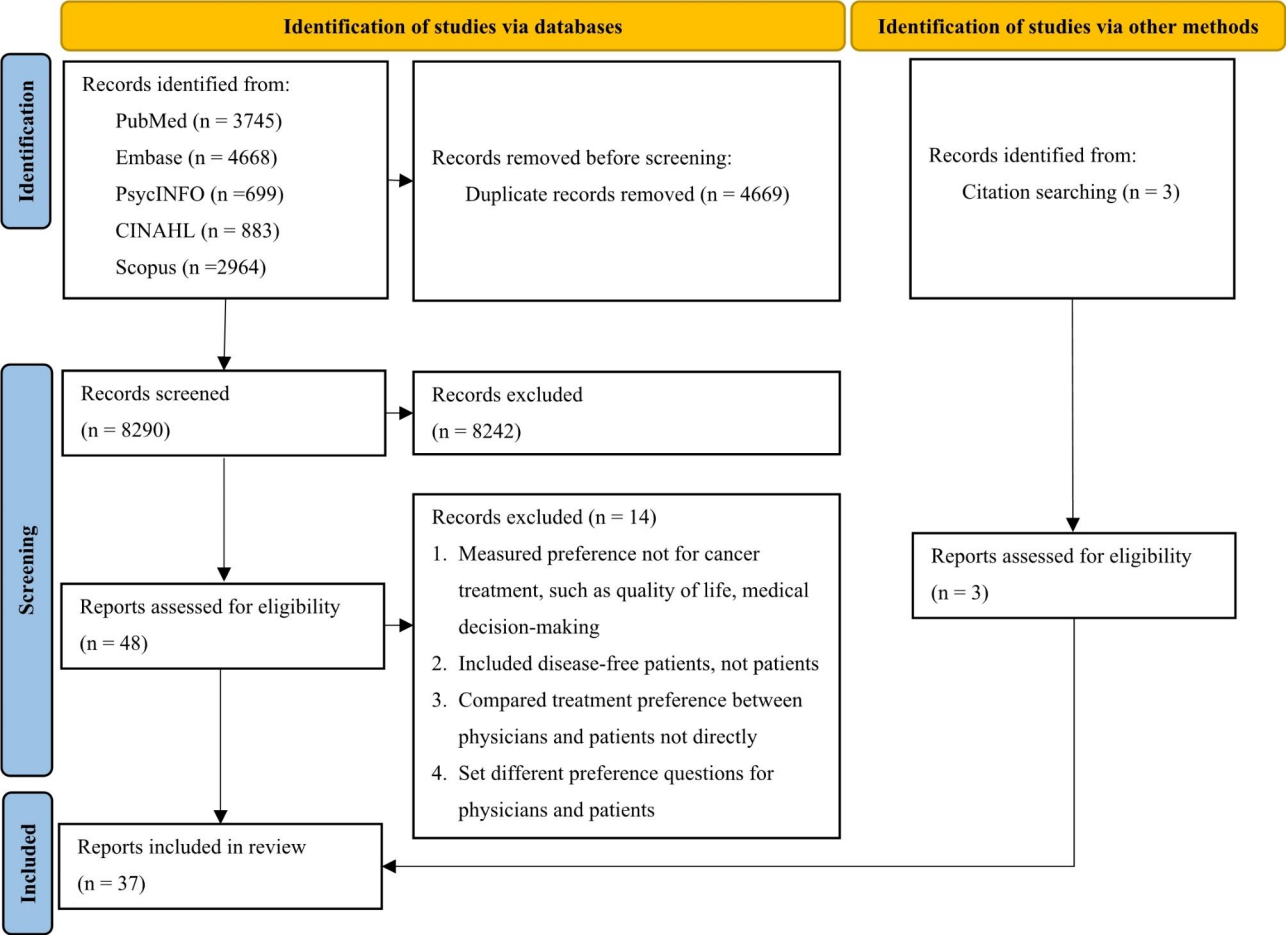
Flow chart of the study selection

The review identified 12,959 publications. After removing 4,669 duplicates, the title and abstract of 8,290 publications were screened for eligibility and 8,242 were excluded. A full-text screening was conducted on the remaining 48 publications, of which 34 were included in the review. Additional 3 studies were included through reviewing the reference lists of identified publications. The detailed selection process is shown in Fig. 1.

In the quality assessment, for DCE and CA studies, the construction of choice tasks and clarification of preference elicitation were often partially reported. For other studies, sample size justification and non-respondent information were often not reported. The final assessment tables were in supplementary Tables 1–2.

### Basic Information

As shown in Table 1, the first study was published in 2003 and the number of studies has increased since 2017 with 11 (30%) conducted in the US. A total of thirteen types of cancer were the target conditions among these studies, including melanoma (n = 6, 16%), breast cancer (n = 6, 16%) and lymphoma (n = 5, 14%). DCE was the most frequently used preference elicitation technique (n = 23, 62%). Convenient samples were most frequently used (n = 20, 54%). The sample size of physicians ranged from 18 to 363 and that of patients varied from 30 to 456. In terms of the mode of administration, online survey was most common (n = 14, 38%), followed by face-to-face (n = 5, 14%) and postal survey (n = 3, 8%). Twelve studies (32%) used two modes, and in ten studies (27%), multiple modes was used for physicians and patients.

**Table 1:** Basic characteristics of included studies

First Author	Year	Country	Cancer type	Elicitation technique	Sample recruitment approach			Sample size		Mode of administration	
					Physicians		Patients	Physicians	Patients	Physicians	Patients
Amin, S.	2022	US	Breast cancer	DCE	Marketing survey company			117	169	Online	
Fernández, O.	2022	Spain	Renal cell carcinoma	DCE	Sponsor company	Hospital		67	105	Online/Paper	
Stellato, D.	2021	Canada	Breast cancer	DCE	Sponsor company	Patient advocacy groups		21	62	Online	
Post, C. C. B.	2021	Netherlands	Endometrial cancer	TTM	Physician groups	Primary physicians		63	171	Online/Paper	
Le, H.	2021	US	Lymphocytic Leukemia	DCE	Marketing survey company	Patient advocacy groups/ physician referral/online communities		151	220	Online	
Beusterien, K.	2021	US	Breast cancer	DCE	Marketing survey company			200	300	Online	
Maculaitis, M. C.	2021	US	Breast cancer	DCE&BWS	Marketing survey company			209	304	Online	
Hauber, B.	2020	US	NSCLC	DCE	Marketing survey company			102	200	Online	
van der Valk, M. J. M.	2020	Netherlands	Rectal cancer	DCE	Hospital			128	94	Online	
Weiss, J.	2020	German	Melanoma	Questionnaire	Hospital			27	30	Postal	
Fifer, S. J.	2019	Australia	Multiple Myeloma	DCE	Specialist healthcare research panels		Patient advocacy groups	28	124	Online	
Stenehjem, D. D.	2019	US	Melanoma	DCE	Registration database			20	233	E-mail/Postal	
Stellato, D.	2019	Canada	Melanoma	DCE	Marketing survey company		Patient advocacy groups	18	39	Online	
Ivanova, J.	2019	US	Soft Tissue Sarcoma	DCE	Marketing survey company		Physician referral/Patient advocate group	160	76	Online	
Nakayama, M.	2018	Japan	Prostate Cancer	DCE	Marketing survey company			127	103	Online	
Gonzalez, J. M.	2018	US	Renal Cell Carcinoma	DCE	Marketing survey company			142	201	Online	
Bröckelmann, P. J.	2018	France, German, UK	Classical Hodgkin Lymphoma	DCE	Marketing survey company			281	289	Online	
Kennedy, E. D.	2018	Canada	Low Rectal Cancer	Threshold Technique	Physician registration database		Hospital	363	50	Postal	Face to face
Kahler, K. C.	2018	German	Melanoma	Threshold Technique	Hospital			108	130	Face to face	
Liu, F. X.	2017	US	Melanoma	DCE	Marketing survey company			150	200	Online	
Lee, J. Y.	2017	Korea	Endometrial Cancer	DCE&TTO	Physician list		Hospital	56	103	Online	Face to face
Gonzalez, J. M.	2017	US	Colorectal Cancer	DCE	Marketing survey company			127	150	Online	
Vaz-Luis, I.	2017	US	Breast Cancer	Questionnaire	Clinical trial			175	456	Online	Telephone/Postal
Pacchiana, M. V.	2017	Italy	NSCLC	Questionnaire	NA			37	92	Face to face	
Landfeldt, E.	2016	German, Sweden	Lymphocytic Leukemia	CA	Marketing survey company		Patient list	72	44	Online	Online/Telephone
Blinman, P.	2016	Australia, New Zealand	Endometrial Cancer	TTO	Clinical trial			44	83	Face to face	
Blinman, P.	2015	Australia	Non-Small-cell Lung Cancer	TTO	Hospital			82	122	Face to face	
Kunneman, M.	2014	Netherlands	Endometrial Cancer	TTM	Physician list		Hospital	77	95	Online	Face to face
Krammer, R.	2014	German	Melanoma	Questionnaire	Hospital			30	30	Face to face	
de Bekker-Grob, E. W.	2013	New Zealand	Prostate Cancer	DCE	Physician list		Hospital	50	110	Postal	
Park, M. H.	2012	Korea	Renal Cell Carcinoma	DCE	Hospital			272	172	E-mail	Face to face
Thrumurthy, S. G.	2011	UK	Esophagogastric Cancer	DCE	Hospital			90	81	Face to face	Postal
Shafey, M.	2011	Canada	Follicular Lymphoma	DCE	Physician list		Hospital	48	81	Postal	
Muhlbacher, A. C.	2011	German	Follicular Lymphoma	DCE	Physician registration database		Patient organization	243	282	E-mail/Postal	
Gandhi, S.	2011	Canada	Breast Cancer	Questionnaire	NA		Hospital	40	153	E-mail	Face to face
Harrison, J. D.	2008	Australia	Rectal Cancer	PMP	Physician association		Hospital	264	103	Postal	Face to face
Solomon, M. J.	2003	Australia	Colorectal Cancer	SG&TTO	Physician association		Hospital	146	110	Postal	Face to face

DCE: Discrete Choice Experiment; TTO: Time Trade-off; TTM: Trade-off Method; PMP: Prospective Measure of Preference Method; SG: Standard Gamble; CA: Conjoint Analysis.

### Attribute identification

Of 24 DCE/CA studies (including 23 DCE studies and 1 CA study), a total of 142 attributes were identified (including duplicated attributes) with 3–8 attributes per study. Various qualitative methods were used to generate the attributes. Twenty studies (54%) developed the attribute list through a literature review and then confirmed them through clinical specialist and/or patient interview. Three studies (8%) only used the interview [18–20], one literature review only [21]; and two (5%) quantitative methods (i.e., principal component analysis, factor analysis and analyses of variance to finalize the attributes) [22, 23]. Sixteen studies (43%) conducted the pilot test to further refine the attributes.

Amongst these attributes, 39 attributes (27%) were about health benefits, 63 adverse effects (44%), 25 treatment process (18%), 5 cost (4%), 8 impact on quality of life (6%), and 2 the provider qualification (1%) (Table 2). All but one study included the attributes of health benefit and adverse effects. Health benefits were commonly measured using survival outcomes, including progression-free survival (PFS, n = 10, 26%), and overall survival (OS, n = 6, 15%). Seven studies (29%) used a generic term for all types of adverse effects such as degree of side effect, and others defined disease-specific adverse effects, including gastrointestinal perforation for kidney cell carcinoma [24] and permanent urinary incontinence for prostate cancer [25]. Mode and frequency of administration, dosing schedule/regimen and further therapies/monitoring were frequently used in the category of treatment process. Five studies (21%) measured the attribute of cost, with three on the cost paid by patients [26–28] and two on the cost by healthcare systems [29, 30]. Six studies (25%) included the attribute about the impact on quality of life. Only one study measured provider qualification [31].

**Table 2:** Classification of attributes in 24 DCE/CA studies

	Health benefit N = 39^*^	Adverse effect N = 63	Treatment process N = 25	Cost N = 5	Impact on quality of life N = 8	Provider qualification N = 2
Amin, S.	Median OS, Median PFS	Risk of neuropathy, Risk of neutropenia, Risk of nausea, Risk of alopecia, Risk of immune-related AE				
Fernández, O.	Progression survival gain	Risk of SAE	Mode of administration	Monthly cost (healthcare system)	HRQoL	
Stellato, D.	Chance of progression-free over 24 months	Improvement in pain, Chance of hot flashes, Chance of neutropenia, Chance of nausea	Dosing regimen, Monitoring			
Le, H.	Chance of 2-year PFS	Risk of atrial fibrillation, Risk of infection, Risk of tumor lysis syndrome, Risk of bleeding, Risk of arthralgia/myalgia/ musculoskeletal pain, Risk of discontinue due to AES	Duration and administration			
Beusterien, K.	Chance of 5-Y invasive DFS	Risk of nausea, Risk of diarrhea, Risk of neutropenia, Risk of alopecia	Dosing schedule, Electrocardiogram monitoring			
Maculaitis, M. C.		Risk of dose reduction due to AES, Risk of diarrhea, Risk of abdominal (belly) pain, Risk of III/IV neutropenia	Regimen, Dosing schedule, Electrocardiogram monitoring			
Hauber, B	Expected survival, Best-case survival, Worst-case survival	Degree of fecal fatigue, Degree of nausea, Risk of febrile neutropenia				
van der Valk, M. J. M.	DFS	Degree of fecal incontinence, Degree of urinary dysfunction, Degree of sexual dysfunction	Further therapies		Worry about cancer recurrence	
Fifer, S. J.	OS, Remission period	Risk of SE	Mode & frequency of administration	Out of pocket (annual)		
Stenehjem, D. D.	OS	Risk of immunotherapy-related SE, Risk of skin toxicity, Risk of gastrointestinal toxicity	Mode of administration	Out of pocket (month)		
Stellato, D.	Chance of cancer-free for 21 months, Chance of free of distant metastases for 21 months, Chance of alive for 36 months	Risk of fever (≥ 39℃), Risk of diarrhea (4–6 episodes daily), Risk of thyroid problems with symptoms	Dosing regimen		Difficulties with work and daily activities	
Ivanova, J.	OS, PFS, ORR	Risk of hospitalization due to SE	Treatment schedule			
Nakayama, M.	Effect to keep disease stable	Degree of SE	Convenience of treatment		QoL	
Gonzalez, J. M.	PFS, 3Y-PL	Degree of skin reactions, Degree of fatigue	Mode & frequency of administration	Co-payment (month)		
Bröckelmann, P. J.	5Y-OS, 5Y-PFS	Risk of SE requiring treatment, Risk of peripheral neuropathy, Risk of infertility, Risk of permanent pulmonary toxicity				
Liu, F. X.	MDT, ORR, PFS, OS	Risk of III/IV SE	Mode of administration, Dosing regimen			
Lee, J. Y.	5Y-recurrence rate	Risk of lymphedema, Surgery-related systemic morbidity				
Gonzalez, J. M.	PFS	Risk of severe papulopustular rash, Risk of serious hemorrhage, Risk of cardiopulmonary arrest				
Landfeldt, E.	OS, PFS	Degree of fatigue, Degree of nausea, Risk of serious infections	Mode & frequency of administration			
de Bekker-Grob, E. W.	Effect of cure	Risk of permanent urinary incontinency, Risk of permanent erectile dysfunction, Risk of other permanent side effects	Frequency of PSA testing with a risk of new prostate biopsies			
Park, M. H.	PFS	Risk of bone marrow suppression, Risk of hand-foot skin reaction, Risk of gastrointestinal perforation, Risk of bleeding	Mode of administration			
Thrumurthy, S. G.	Mortality, Morbidity, Cure rate				QoL	Hospital type, Surgeon’s reputation
Shafey, M.	Median PFS & 5Y-PFS	Degree of SE	Mode & frequency of administration	Health cost (healthcare system)		
Muhlbacher, A. C.	Increase in life-span	Degree of SE	Further therapies, Self-medication, Breaks in treatment		Emotional situation, Physical situation, Social situation	

QoL, Quality of Life; HRQoL, Health related quality of life, SE, Side Effect; AE, Adverse effect, SAE, Serious adverse effect, PFS, Progression-free Survival; 3Y-PL, Probability of Living at Least 3 Years; 5Y-OS, 5 Years Overall Survival; 5Y-PFS, 5 Years Progression-free Survival; MDT, Median Duration of Therapy; ORR, Objective Response Rate; OS, Overall Survival; III/IV SE, III/IV side effects

* N was the number of attributes

Questions in non-DCE/CA studies were mostly self-developed based on clinical evidence and pretested. They focused on two main categories, namely health benefit and adverse effects, where health benefit was more about survival rate and life years, while adverse effects were about the risk of cancer recurrence. For instance, physicians and patients were asked to consider how much the extra chance of survival or the potential risk of local regrowth was, a certain treatment could be accepted [32, 33].

### Concordances and discordances in preference between physicians and patients

**Fig. 2:**
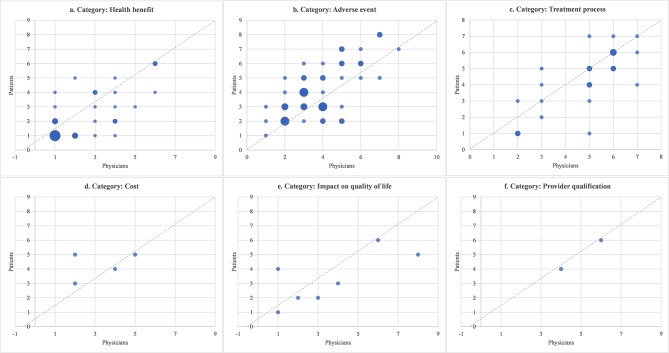
Relative rank of every attribute in 20 DCE/CA studies. Note: Every scatter indicates an attribute and the size of it is proportional to the number of studies with corresponding ranks

The ranks of attributes by physicians vs. patients in 20 DCE/CA studies are plotted in Fig. 2 (the original ranks were shown in supplementary Table 3). Among all categories, health benefit was valued most with the first rank by both physicians and patients (n = 10, 50%). Treatment process, cost and provider qualification were less important indicated by lower ranks.

**Table 3:** Threshold scores in 8 threshold technique/TTO/TTM studies

	Preferences measurement question	Objective point	Threshold scores		Consistency (P-value)
			Physicians	Patients	
Post, C. C. B.	1 Accept chemoradiotherapy VS. radiotherapy alone	5Y-survival rate (over baseline rate)	5%	10%	0.024
Kennedy, E. D.	1 Accept nonoperative management VS. abdominal perineal resection	5Y-local regrowth rate	5.0%	20.0%	NA
	2 Accept nonoperative management VS. abdominal perineal resection	5Y-survival rate	75.0%	60.0%	NA
Kahler, K. C.	1 Accept interferon alfa-2a and 2b (IFN) with mild-to-moderate side effects VS. without any IFN treatment	5Y-DFS rate	42.3%	59.6%	< 0.001
	2 Accept interferon alfa-2a and 2b (IFN) with severe side effects VS. without any IFN treatment		53.6%	69.8%	< 0.001
	3 Accept mild-to-moderate side effects for adjuvant IFN	Risk of recurrence	46.0%	65.0%	0.001
Lee, J. Y.	1 Accept no lymphadenectomy VS. routine lymphadenectomy	Risk of recurrence	2.75%	3%	0.620
Blinman, P.	1 Accept the addition of adjuvant chemotherapy to pelvic radiotherapy VS. no treatment (5 life years)	Life years	6 years	6 years	0.400
	2 Accept the addition of adjuvant chemotherapy to pelvic radiotherapy VS. no treatment (8 life years)		9 years	9 years	
	3 Accept the addition of adjuvant chemotherapy to pelvic radiotherapy VS. no treatment (50% survival rate at 5 years)	Survival rate	57%	55%	0.030
	4 Accept the addition of adjuvant chemotherapy to pelvic radiotherapy VS. no treatment (65% survival rate at 5 years)		75%	69%	
Blinman, P.	1 Accept the adjuvant chemotherapy VS. no treatment (3 life years)	Life years	3.9 years	3.9 years	NA
	2 Accept the adjuvant chemotherapy VS. no treatment (5 life years)		5.9 years	5.9 years	
	3 Accept the adjuvant chemotherapy VS. no treatment (50% survival rate at 5 years)	Survival rate	55%	55%	
	4 Accept the adjuvant chemotherapy VS. no treatment (65% survival rate at 5 years)		70%	70%	
Kunneman, M.	1 Accept surgery only VS. vaginal brachy therapy and surgery	5Y-recurrence rate	10%	2%	< 0.001
Solomon, M. J. (TTO Part)	1 Accept laparoscopic surgery VS. open surgery	Mortality risk	3.20%	5%	NA
	2 Accept local resection VS. colostomy		13.40%	17.20%	
	3 Accept surgery alone VS. surgery and chemotherapy		12.70%	21.40%	
	4 Accept chemoradiotherapy and no surgery VS. surgery and colostomy		16.65%	6.10%	

The preferences measurement questions refer to the trade-offs between two listed treatments. Taking the example of the Post’s study, the question would be “the desired 5-year overall survival benefit over the baseline rate to accept chemoradiotherapy relative to radiotherapy alone”. Then the value to this question, namely, the threshold score of physicians was 5%, lower than the corresponding rate of patients as 10%.

Among 20 attributes of health benefit with different ranks from patients and physicians, patients gave higher ranks in 11 attributes (55%) than their physicians did, including OS, PFS, ORR, cure rate, remission period, morbidity, chance of cancer-free and chance of distant metastases free. Patients also gave higher ranks among 11 out of 16 attributes (69%) for treatment process. While physicians placed higher importance on adverse effects. Among 38 attributes of adverse effects with different ranks, physicians valued 25 attributes (66%) higher than patients did. Due to the limited number of attributes on cost, impact on quality of life and provider qualification, no meaningful difference could be summarized.

Of 8 threshold technique/TTO/TTM studies (Table 3), one study showed the same threshold scores for both physicians and patients [34], and one study showed mixed results that physicians and patients held the same threshold scores towards life years but different scores towards survival rate [32]. The remaining 6 studies all showed discordance between physicians and patients with 3 reaching statistical significance [35–37]. Among 5 studies measuring the trade-off towards the risk of regrowth rate or recurrence rate, 4 studies reported higher threshold scores in patients than physicians [33, 36–39]. Among 4 studies that valued the minimum survival to accept the treatment and with different threshold scores, patients had higher scores than physicians in two studies [35, 36] while lower in the other two [32, 33].

Five self-designed questionnaire studies showed physicians expected more health benefits from treatment, while patients rather accepted a treatment even with smaller health benefits (Table 4). Taking the example of Vaz-Luis’s study, only 18% of physicians considered 6 months of chemotherapy worthwhile for 1-month survival benefit, while 42% of patients considered so [40]. In addition, Krammer et al. showed that patients and physicians differed in their trade-off between survival and side effects [41]. When choosing from 16 weeks survival with moderate side effects and 8 weeks survival with mild side effects, 83% of physicians preferred the former, while 56% of patients did so [41]. Similarly, the willingness to trade in 2 SG/PMP studies revealed that patients preferred to use more remaining life years to avoid the treatment risk and treatment-related side effect on daily life (Table 5) [39, 42].

**Table 4:** Results of 5 questionnaire studies

	Preferences measurement question	Proportion		Consistency (P-value)
		Physicians	Patients	
Weiss, J.	1 19 months living with combination immunotherapy and severe side effects in 36% VS. 9 months living with standard immunotherapy and severe side effects in 15%	70%/30%	45%/55%	0.050
	2 12 months living with combination immunotherapy and severe side effects in 36% VS. 11 months living with standard immunotherapy and severe side effects in 15%	15%/85%	17%/83%	NA
	3 24 months living with combination immunotherapy and severe side effects in 36% VS. 3 months of pain-free living without tumor therapy with palliative therapy	80%/20%	50%/50%	0.018
	4 Agree to a treatment with many side effects at any time and with the very low prospect of prolonging life	60%	30%	NA
	5 Prefer to receive the infusions every three weeks rather than every two weeks with the equivalent effect	92%	83%	
	6 Prefer early palliative therapy to a therapy rich in side effects if there is no prospect of healing	59%	57%	
Vaz-Luis, I.	1 Whether 6 months of chemotherapy would be worthwhile for a 1-, 2-, 6-, 9-, 12-, and 24-month survival benefit	1-month: 18% 2-month: 37% 6-month: 86% 9-month: 93% 12-month: 97% 24-month: 97%	1-month: 42% 2-month: 57% 6-month: 79% 9-month: 87% 12-month: 93% 24-month: 96%	NA
Pacchiana, M. V.	1 Whether interested in maintenance therapy, rather than treatment-free	97%	75%	0.003
	2 Whether interested in maintenance therapy if improving life expectancy by about 1 Month, 3 Month, 6 Month, 1 Year	1-month: 14% 3-month: 62% 6-month: 89% 1 Year: 100%	1-month: 46% 3-month: 61% 6-month: 76% 1 Year: 88%	1-month: <0.001 3-month: 0.910 6-month: 0.080 1 Year: 0.030
	3 Whether interested in maintenance therapy if providing no survival benefit but would result in symptom control	78%	74%	0.630
	4 Whether interested in maintenance therapy if providing no survival benefit but would result in radiologic tumor stabilization	38%	62%	0.010
Krammer, R.	1 16 weeks survival with moderate side effects with ipilimumab VS. 8 weeks survival with mild side effects with chemotherapy	83%/17%	56%/44%	NA
	2 3 months survival with mild side effects with chemotherapy VS. 3 months survival free of symptoms with palliative care	10%/90%	32%/68%	
	3 Spending €100.000 for ipilimumab VS. palliative care VS. skin screening VS. primary prevention	3%/21%/10%/66%	4%/4%/46%/46%	
Gandhi, S.	1 Minimum overall survival required to continue aromatase inhibitor 5 years	<1%: 0% 1–2%: 45% 2–5%: 37.5% 5–10%: 12.5% 10–15%: 0% 15–20%: 2.5% >20%: 0%	<1%: 30.1% 1–2%: 14.4% 2–5%: 11.8% 5–10%: 12.4% 10–15%: 3.9% 15–20%: 3.9% >20%: 17.0%	NA
	2 Minimum decrease in risk of cancer recurrence required to continue aromatase inhibitor 5 years	<1%: 0% 1–2%: 2.5% 2–5%: 37.5% 5–10%: 35.0% 10–15%: 12.5% 15–20%: 0% >20%: 2.5%	<1%: 27.5% 1–2%: 14.4% 2–5%: 13.1% 5–10%: 14.4% 10–15%: 4.6% 15–20%: 5.9% >20%: 14.4%	

**Table 5:** Willingness-to-trade in 2 SG/PMP studies

		Preferences measurement question	Willingness-to-trade		Consistency (P-value)
			Physicians	Patients	
Harrison, J. D.	Proportion of remaining life expectancy could be traded to	1 Avoid abdominoperineal resection	14.3%	34.0%	NA
		2 Avoid anterior resection and chemoradiotherapy	9.7%	24.0%	
		3 Avoid anterior resection and chemotherapy	8.0%	20.0%	
		4 Avoid anterior resection and preoperative radiotherapy	8.3%	17.0%	
		5 Avoid anterior resection and postoperative radiotherapy	12.7%	20.0%	
Solomon, M. J. (SG Part)		1 Accept laparoscopic surgery relative to open surgery	1.50%	0.80%	NA
		2 Accept local resection relative to colostomy	9.50%	2.70%	
		3 Accept surgery alone relative to surgery and chemotherapy	5.85%	2.50%	
		4 Accept chemoradiotherapy and no surgery relative to surgery and colostomy	9.35%	0.80%	

## Discussion

This study systematically reviewed the discrepancies in preferences for cancer treatment between physicians and patients. Health benefit and adverse effects were key drivers of treatment preferences, and the trade-offs between them were the primary focus of the included studies. Compared to physicians, patients valued health benefit more and were willing to take on more risks of adverse effects. Patients also placed a higher weight on the treatment process than physicians did. The preference differences between physicians and patients varied across studies.

Current preference measurement studies focused on the trade-off between health benefit and adverse effects [16, 43]. Existing threshold technique, such as TTO, TTM, SG or PMP, could only evaluate the tradeoffs between two attributes. Although DCE or CA can include more attributes, achieving clinical relevance might require detailed attributes, thereby increase the possible combinations of all attributes and the complexity of the experiment design, which could discourage researchers [44]. Further studies could consider other methods that can incorporate more attributes and be flexible in supporting real-world decisions. For example, adaptive conjoint analysis can include more attributes and customize the preference elicitation based on prior responses [45].

An important difference was that generally, patients placed a higher value on health benefit and physicians on adverse effects. As health care providers, physicians are process-oriented and focus on the whole treatment including safety. In contrast, patients are result-oriented, for whom survival benefit is the most important. The included studies showed that whether in active treatment aiming to keep functioning in the long term, or adjuvant treatment or maintenance therapy aiming to lower cancer recurrence risk, patients always expect survival benefits. Moreover, patients often preferred to seek active treatment and wanted to make sure they have tried every treatment option [46, 47]. Patients also tended to behave as risk-takers and overlooked the concerns of having adverse effects [48]. When seeking treatments, patients may assume their own survival gains are more favorable and exceed the population average gain [49, 50]. This “value of hope” also drives them to accept a higher risk [51]. While adverse effects of cancer treatment may have non-negligible impact on patient preferences [39, 42]. For some patients, survival is preferred but conditional upon no worsening in quality of life [52]. Prior longitudinal research also found that patients with advanced cancer placed stronger emphasis on quality of life (vs. survival) as the treatment goal [53].

Patients also concerned more on treatment process than physicians, which were closely related to their daily life. The review conducted by Harrison et al. also reported that medication safety, delivery and timing of treatment, and treatment accessibility were more important to patients [10]. As a part of patient experience, treatment process is one of the most common indicators used to evaluate the healthcare services [54].

The differences in treatment preferences between physicians and patients varied across various studies. For example, both Stellato’s and Liu’s studies examined the preferences for melanoma treatment, and Stellato et al. concluded that the physician and patient had the same preferences [55], while Liu et al., showed physicians valued adverse effect most while patients valued survival most [56]. Even in DCE or CA studies, physician and patient preferences for the same attribute had heterogeneity across studies. Studies by Gonzalez et al., Brockelmann et al. and Park et al. all showed that physicians valued PFS more than patients [24, 28, 57], while the study by Landfeldt et al. indicated that patients valued PFS more [58]. And Liu et al. showed that physicians and patients weighed PFS similarly [56]. The preference differences may be correlated with the individual characteristics. Current preference studies focused on the aggerated level that revealed the sample average preference, other than the individual level and personal preference. Individual preference heterogeneity is remaining a salient topic [59]. The preference differences may be also impacted by patient’s treatment experience. Patients who have survived or recovered from previous treatments may develop positive experiences about the treatment and therefore tend to favor the choice they were offered rather than the alternative [60, 61]. This generally resulted from normal psychologic processes called cognitive dissonance reduction and adaptation mechanisms [62]. Another possible explanation for this difference could be that in different studies, patients and physicians had different understandings of the survival or risk statistics in the questions. Using standard decision aids and consistent illustrations for statistics might help form the common understanding and increase the comparability across the studies.

The difference in cancer treatment preferences between physicians and patients may have important implications on treatment decision making. As physicians and patients are mainly concern about the benefit-risk trade-off, and always have different preferences on it, evidence on these two attributes should be carefully discussed in SDM [63, 64]. Further to facilitate SDM, physicians may also need to master the ability of communicating evidence in a clear, understandable, and non-misleading manner [65]. Training physicians with sufficient SDM knowledge or skill is essential [66]. In addition, some tools have been developed to assist physicians with implementing SDM into their practice, like SHARE approach developed by the AHRQ (Agency for Healthcare Research and Quality) [67]. Moreover, patients should be encouraged to actively convey preferences and understand the importance of their participation [68]. The educational material could be distributed to improve the awareness and importance of SDM among patients [66]. Furthermore, the development of clinical practice guidelines that should take into account the discordance in preference between physicians and patients and discuss the implications on SDM.

There are some limitations in this review. First, qualitative studies were excluded. Second, non-quantitative synthesis was done due to the heterogeneity of included studies.

## Conclusion

This review found that there were important differences in treatment preferences between physicians and cancer patients. Patients placed a higher weight on health benefit and treatment process, while physicians placed higher weight on adverse effects.

### Electronic supplementary material

Below is the link to the electronic supplementary material.

Supplementary Material 1

Supplementary Material 2

## Data Availability

The data analyzed during the current study available from the corresponding author on reasonable request.

## Abbreviations

SDM: Shared decision-making
DCE: Discrete choice experiment
PRISMA: Preferred Reporting Items for Systematic Reviews and Meta-Analyses
CA: Conjoint analysis
TTO: Time trade-off
TTM: Trade-off method
SG: Standard gamble
PMP: Prospective measure of preference
ISPOR: International Society for Pharmacoeconomics and Outcomes Research
AXIS: Appraisal Tool for Cross-Sectional Studies
AHRQ: Agency for Healthcare Research and Quality
