# Supplementary material for: Differences between physician and patient preferences for cancer treatments: a systematic review
Source: BMC Cancer. 2023 Nov 18;23:1126. doi: 10.1186/s12885-023-11598-4 (PMC10657542; doi:10.1186/s12885-023-11598-4)
Supplement: Supplementary file 1 — Supplementary Material 1 [file 12885_2023_11598_MOESM1_ESM.docx]

**Appendix 1 Search Strategy**

Database: PubMed

Search Strategy:

1. "neoplasms"[MeSH Terms]
2. "tumor*"[Title/Abstract] OR "neoplam*"[Title/Abstract] OR "cancer*"[Title/Abstract] OR "oncology"[Title/Abstract] OR "malignan*"[Title/Abstract]
3. #1 OR #2
4. "physician*"[Title/Abstract] OR "doctor*"[Title/Abstract] OR "clinician*"[Title/Abstract] OR "mediciner*"[Title/Abstract] OR "oncologist*"[Title/Abstract] OR "provider*"[Title/Abstract]
5. "patient*"[Title/Abstract]
6. "preference*"[Title/Abstract]
7. #3 AND #4 AND #5 AND #6

Database: Embase

Search Strategy:

1. 'neoplasms'/exp OR neoplasms
2. tumor* OR neoplam* OR cancer* OR oncology OR malignan*:ab,ti
3. #1 OR #2
4. physician* OR doctor* OR clinician* OR mediciner* OR oncologist* OR provider*:ab,ti
5. patient*:ab,ti
6. preference*:ab,ti
7. #3 AND #4 AND #5 AND #6

Database: PsycInfo/CINAHL (via EBSCO host)

Search Strategy:

1. TI ( tumor* OR neoplam* OR cancer* OR oncology OR malignan* ) OR AB ( tumor* OR neoplam* OR cancer* OR oncology OR malignan* )
2. TI ( physician* OR doctor* OR clinician* OR mediciner* OR oncologist* OR provider* ) OR AB ( physician* OR doctor* OR clinician* OR mediciner* OR oncologist* OR provider* )
3. TI patient* OR AB patient*
4. TI preference* OR AB preference*
5. S1 AND S2 AND S3 AND S4

Database: Scopus

Search Strategy:

1. TITLE-ABS ( tumor* OR neoplam* OR cancer* OR oncology OR malignan* )
2. TITLE-ABS ( physician* OR doctor* OR clinician* OR mediciner* OR oncologist* OR provider* )
3. TITLE-ABS ( patient* )
4. TITLE-ABS ( preference* )
5. #1 AND #2 AND #3 AND #4 AND ( LIMIT-TO ( LANGUAGE , "English" ) ) AND ( LIMIT-TO ( DOCTYPE , "ar" ) )

**Appendix 2 ISPOR Report: A Checklist for Conjoint Analysis Applications in Health Care**

1. Was a well-defined research question stated and is conjoint analysis an appropriate method for answering it?

1.1 Were a well-defined research question and a testable hypothesis articulated?

1.2 Was the study perspective described, and was the study placed in a particular decision-making or policy context?

1.3 What is the rationale for using conjoint analysis to answer the research question?

2. Was the choice of attributes and levels supported by evidence?

2.1 Was attribute identification supported by evidence (literature reviews, focus groups, or other scientific methods)?

2.2 Was attribute selection justified and consistent with theory?

2.3 Was level selection for each attribute justified by the evidence and consistent with the study perspective and hypothesis?

3. Was the construction of tasks appropriate?

3.1 Was the number of attributes in each conjoint task justified (that is, full or partial profile)?

3.2 Was the number of profiles in each conjoint task justified?

3.3 Was (should) an opt-out or a status-quo alternative (be) included?

4. Was the choice of experimental design justified and evaluated?

4.1 Was the choice of experimental design justified? Were alternative experimental designs considered?

4.2 Were the properties of the experimental design evaluated?

4.3 Was the number of conjoint tasks included in the data-collection instrument appropriate?

5. Were preferences elicited appropriately, given the research question?

5.1 Was there sufficient motivation and explanation of conjoint tasks?

5.2 Was an appropriate elicitation format (that is, rating, ranking, or choice) used? Did (should) the elicitation format allow for indifference?

5.3 In addition to preference elicitation, did the conjoint tasks include other qualifying questions (for example, strength of preference, confidence in response, and other methods)?

6. Was the data collection instrument designed appropriately?

6.1 Was appropriate respondent information collected (such as sociodemographic, attitudinal, health history or status, and treatment experience)?

6.2 Were the attributes and levels defined, and was any contextual information provided?

6.3 Was the level of burden of the data-collection instrument appropriate? Were respondents encouraged and motivated?

7. Was the data-collection plan appropriate?

7.1 Was the sampling strategy justified (for example, sample size, stratification, and recruitment)?

7.2 Was the mode of administration justified and appropriate (for example, face-to-face, pen-and-paper, web-based)?

7.3 Were ethical considerations addressed (for example, recruitment, information and/or consent, compensation)?

8. Were statistical analyses and model estimations appropriate?

8.1 Were respondent characteristics examined and tested?

8.2 Was the quality of the responses examined (for example, rationality, validity, reliability)?

8.3 Was model estimation conducted appropriately? Were issues of clustering and subgroups handled appropriately?

9. Were the results and conclusions valid?

9.1 Did study results reflect testable hypotheses and account for statistical uncertainty?

9.2 Were study conclusions supported by the evidence and compared with existing findings in the literature?

9.3 Were study limitations and generalizability adequately discussed?

10. Was the study presentation clear, concise, and complete?

10.1 Was study importance and research context adequately motivated?

10.2 Were the study data-collection instrument and methods described?

10.3 Were the study implications clearly stated and understandable to a wide audience?

**Appendix 3** **The Appraisal tool for Cross-Sectional Studies (AXIS)**

Introduction

1. Were the aims/objectives of the study clear?

Methods

2. Was the study design appropriate for the stated aim(s)?

3. Was the sample size justified?

4. Was the target/reference population clearly defined? (Is it clear who the research was about?)

5. Was the sample frame taken from an appropriate population base so that it closely represented the target/reference population under investigation?

6. Was the selection process likely to select subjects/participants that were representative of the target/reference population under investigation?

7. Were measures undertaken to address and categorise non-responders?

8. Were the risk factor and outcome variables measured appropriate to the aims of the study?

9. Were the risk factor and outcome variables measured correctly using instruments/ measurements that had been trialled, piloted or published previously?

10. Is it clear what was used to determined statistical significance and/or precision estimates? (eg, p values, CIs)

11. Were the methods (including statistical methods) sufficiently described to enable them to be repeated?

Results

12. Were the basic data adequately described?

13. Does the response rate raise concerns about non-response bias?

14. If appropriate, was information about non-responders described?

15. Were the results internally consistent?

16. Were the results for the analyses described in the methods, presented?
