# Supplementary material for: Differences between physician and patient preferences for cancer treatments: a systematic review
Source: BMC Cancer. 2023 Nov 18;23:1126. doi: 10.1186/s12885-023-11598-4 (PMC10657542; doi:10.1186/s12885-023-11598-4)
Supplement: Supplementary file 2 — Supplementary Material 2 [file 12885_2023_11598_MOESM2_ESM.docx]

Supplementary Table.1 Appraisal and quality assessment for DCE/CA studies

|  | Well-defined research question stated | Choice of attributes and levels supported by evidence | Construction of tasks appropriate | Choice of experimental design justified and evaluated | Preferences elicited appropriately | Data collection instrument designed appropriately | Data-collection plan appropriate | Statistical analyses and model estimations appropriate | Results and conclusions valid | Study presentation clear, concise, and complete |
| --- | --- | --- | --- | --- | --- | --- | --- | --- | --- | --- |
| Amin, S. 2022 | Yes | Yes | Yes | Yes | Partial | Yes | Yes | Yes | Yes | Yes |
| Fernández, O. 2022 | Yes | Partial | Partial | Yes | Partial | Yes | Yes | Yes | Yes | Yes |
| Stellato, D. 2021 | Yes | Partial | Yes | Yes | Partial | Yes | Yes | Yes | Yes | Yes |
| Le, H. 2021 | Yes | Partial | Partial | Yes | Partial | Yes | Yes | Yes | Yes | Yes |
| Beusterien, K. 2021 | Yes | Partial | Unknown | Partial | Partial | Yes | Yes | Yes | Yes | Yes |
| Maculaitis, M. C. 2021 | Yes | Yes | Partial | Yes | Yes | Yes | Yes | Yes | Yes | Yes |
| Hauber, B. 2020 | Yes | Partial | Partial | Partial | Partial | Yes | Yes | Yes | Yes | Yes |
| van der Valk, M. J. M. 2020 | Yes | Partial | Yes | Partial | Yes | Yes | Yes | Yes | Yes | Yes |
| Fifer, S. J. 2019 | Yes | Yes | Yes | Partial | Partial | Yes | Yes | Yes | Yes | Yes |
| Stenehjem, D. D. 2019 | Yes | Yes | Yes | Yes | Yes | Yes | Yes | Yes | Yes | Yes |
| Stellato, D. 2019 | Yes | Yes | Yes | Yes | Partial | Yes | Yes | Yes | Yes | Yes |
| Ivanova, J. 2019 | Yes | Partial | Partial | Yes | Partial | Yes | Yes | Yes | Yes | Yes |
| Nakayama, M. 2018 | Yes | Yes | Unknown | Partial | Partial | Unknown | Yes | Yes | Yes | Partial |
| Gonzalez, J. M. 2018 | Yes | Yes | Partial | Yes | Partial | Partial | Yes | Yes | Yes | Yes |
| Bröckelmann, P. J. 2018 | Yes | Yes | Yes | Partial | Partial | Yes | Yes | Yes | Yes | Yes |
| Liu, F. X. 2017 | Yes | Yes | Partial | Yes | Yes | Yes | Yes | Yes | Yes | Yes |
| Lee, J. Y. 2017 | Yes | Partial | Partial | Partial | Yes | Yes | Yes | Yes | Yes | Yes |
| Gonzalez, J. M. 2017 | Yes | Yes | Partial | Yes | Partial | Yes | Yes | Yes | Yes | Yes |
| Landfeldt, E. 2016 | Yes | Yes | Partial | Yes | Partial | Yes | Yes | Yes | Yes | Yes |
| de Bekker-Grob, E. W. 2013 | Yes | Yes | Yes | Yes | Partial | Yes | Yes | Yes | Yes | Yes |
| Park, M. H. 2012 | Yes | Yes | Partial | Yes | Yes | Yes | Yes | Yes | Yes | Yes |
| Thrumurthy, S. G. 2011 | Yes | Partial | Yes | Yes | Yes | Yes | Yes | Yes | Yes | Yes |
| Shafey, M. 2011 | Yes | Partial | Partial | Yes | Partial | Yes | Yes | Yes | Yes | Yes |
| Muhlbacher, A. C. 2011 | Yes | Yes | Unknown | Partial | Yes | Yes | Yes | Yes | Yes | Yes |

DCE, Discrete Choice Experiment; CA, Conjoint Analysis

Supplementary Table.2 Appraisal and quality assessment for other studies except DCE/CA studies

|  | Aims | Study design | Sample size | Target population | Sample frame | Selection process | address non-responders | Risk factor and outcome^*^ | Statistical significance | Methods | Basic data | Response rate | Information about non-responders | Results internally consistent | Results for the analyses described in the methods presented |
| --- | --- | --- | --- | --- | --- | --- | --- | --- | --- | --- | --- | --- | --- | --- | --- |
| Post, C. C. B. 2021 | Yes | Yes | No | Yes | Yes | Yes | No | Not applicable | Yes | Yes | Yes | No | No | Yes | Yes |
| Weiss, J. 2020 | Yes | Yes | No | Yes | Yes | Yes | No | Not applicable | Yes | Yes | Yes | No | No | Yes | Yes |
| Kennedy, E. D. 2018 | Yes | Yes | Yes | Yes | Yes | Yes | No | Not applicable | Yes | Yes | Yes | Yes | Yes | Yes | Yes |
| Kahler, K. C. 2018 | Yes | Yes | No | Yes | Yes | Yes | No | Not applicable | Yes | Yes | Yes | No | No | Yes | Yes |
| Vaz-Luis, I. 2017 | Yes | Yes | No | Yes | Yes | Yes | No | Not applicable | Yes | Yes | Yes | No | No | Yes | Yes |
| Pacchiana, M. V. 2017 | Yes | Yes | No | Yes | Yes | Yes | No | Not applicable | Yes | Yes | Yes | Do not comment | No | Yes | Yes |
| Blinman, P. 2016 | Yes | Yes | No | Yes | Yes | Yes | No | Not applicable | Yes | Yes | Yes | No | Yes | Yes | Yes |
| Blinman, P. 2015 | Yes | Yes | Yes | Yes | Yes | Yes | No | Not applicable | Yes | Yes | Yes | No | No | Yes | Yes |
| Kunneman, M. 2014 | Yes | Yes | No | Yes | Yes | Yes | No | Not applicable | Yes | Yes | Yes | Yes | No | Yes | Yes |
| Krammer, R. 2014 | Yes | Yes | No | Yes | Yes | Yes | No | Not applicable | Yes | Yes | Yes | Do not comment | No | Yes | Yes |
| Gandhi, S. 2011 | Yes | Yes | No | Yes | Yes | Yes | No | Not applicable | No | Yes | Yes | Do not comment | No | Yes | Yes |
| Harrison, J. D. 2008 | Yes | Yes | Yes | Yes | Yes | Yes | No | Not applicable | Yes | Yes | Yes | No | Yes | Yes | Yes |
| Solomon, M. J. 2003 | Yes | Yes | Yes | Yes | Yes | Yes | No | Not applicable | Yes | Yes | Yes | No | No | Yes | Yes |

DCE, Discrete Choice Experiment; CA, Conjoint Analysis

*Risk factor and outcome includes item 8 and 9 in the Appraisal tool for Cross-Sectional Studies (AXIS)

Supplementary Table.3 Relative rank of every attribute in 20 DCE/CA studies

|  |  | Attributes/Relative rank of every attribute | | | | | | | |
| --- | --- | --- | --- | --- | --- | --- | --- | --- | --- |
| Amin, S. |  | median OS | median PFS | risk of neuropathy | risk of neutropenia | risk of nausea | risk of alopecia | risk of immune-related AE |  |
|  | Physicians | 1 | 3 | 2 | 5 | 4 | 6 | 7 |  |
|  | Patients | 1 | 4 | 3 | 7 | 2 | 6 | 5 |  |
| Fernández, O. |  | progression survival gain | risk of SAE | mode of administration | monthly cost | HRQoL |  |  |  |
|  | Physicians | 1 | 4 | 3 | 5 | 2 |  |  |  |
|  | Patients | 1 | 3 | 4 | 5 | 2 |  |  |  |
| Stellato, D. |  | chance of progression-free over 24 months | improvement in pain | chance of hot flashes | chance of neutropenia | chance of nausea | dosing regimen | monitoring |  |
|  | Physicians | 1 | 2 | 4 | 5 | 6 | 7 | 3 |  |
|  | Patients | 1 | 4 | 5 | 2 | 7 | 6 | 3 |  |
| Le, H. |  | chance of 2-year PFS | risk of atrial fibrillation | risk of infection | risk of tumor lysis syndrome | risk of bleeding | risk of arthralgia/ myalgia/musculoskeletal pain | risk of discontinue due to AEs | duration and administration |
|  | Physicians rank | 1 | 2 | 4 | 8 | 7 | 5 | 3 | 6 |
|  | Patients rank | 1 | 3 | 2 | 7 | 8 | 6 | 4 | 5 |
| Beusterien, K. |  | chance of 5-Y invasive DFS | risk of nausea | risk of diarrhea | risk of neutropenia | risk of alopecia | dosing schedule | electrocardiogram monitoring |  |
|  | Physicians rank | 1 | 3 | 2 | 4 | 5 | 6 | 7 |  |
|  | Patients rank | 1 | 4 | 2 | 3 | 5 | 6 | 7 |  |
| Maculaitis, M. C. |  | risk of dose reduction due to AEs | risk of diarrhea | risk of abdominal (belly) pain | risk of Ⅲ/Ⅳ neutropenia | regimen | dosing schedule | electrocardiogram monitoring |  |
|  | Physicians rank | 4 | 1 | 3 | 2 | 6 | 7 | 5 |  |
|  | Patients rank | 5 | 1 | 3 | 2 | 6 | 4 | 7 |  |
| Hauber, B. |  | expected survival | best-case survival | worst-case survival | degree of fatigue | degree of nausea | risk of febrile neutropenia |  |  |
|  | Physicians rank | 1 | 2 | 6 | 3 | 5 | 4 |  |  |
|  | Patients rank | 4 | 1 | 6 | 5 | 2 | 3 |  |  |
| van der Valk, M. J. M. |  | DFS | degree of fecal incontinence | degree of urinary dysfunction | degree of sexual dysfunction | further therapies | worry about cancer recurrence |  |  |
|  | Physicians rank | 6 | 2 | 4 | 3 | 5 | 1 |  |  |
|  | Patients rank | 6 | 2 | 3 | 5 | 1 | 4 |  |  |
| Fifer, S. J. |  | OS | remission period | risk of SE | administration | out of pocket costs (annual) |  |  |  |
|  | Physicians rank | 1 | 4 | 3 | 5 | 2 |  |  |  |
|  | Patients rank | 1 | 3 | 2 | 4 | 5 |  |  |  |
| Stenehjem, D. D. |  | OS | risk of immunotherapy-related side effect | risk of Skin toxicity | out of pocket (month) |  |  |  |  |
|  | Physicians rank | 1 | 2 | 3 | 4 |  |  |  |  |
|  | Patients rank | 1 | 2 | 3 | 4 |  |  |  |  |
| Stellato, D. |  | chance of cancer-free for 21 months | chance of free of distant metastases for 21 months | chance of alive for 36 months | risk of fever (≥39℃) | risk of diarrhea (4–6 episodes daily) | risk of thyroid problems with symptoms | dosing regimen | difficulties with work and daily activities |
|  | Physicians rank | 3 | 6 | 1 | 7 | 4 | 5 | 2 | 8 |
|  | Patients rank | 1 | 4 | 2 | 8 | 6 | 7 | 3 | 5 |
| Ivanova, J. |  | OS | PFS | ORR | risk of hospitalization due to SE | treatment schedule |  |  |  |
|  | Physicians rank | 1 | 3 | 4 | 2 | 5 |  |  |  |
|  | Patients rank | 1 | 4 | 2 | 3 | 5 |  |  |  |
| Nakayama, M. |  | effect to keep disease stable | degree of SE | convenience of treatment | QoL |  |  |  |  |
|  | Physicians rank | 1 | 4 | 2 | 3 |  |  |  |  |
|  | Patients rank | 3 | 3 | 1 | 2 |  |  |  |  |
| Gonzalez, J. M. |  | PFS | 3Y-PL | degree of skin reactions | degree of fatigue | mode & frequency of administration | co-payment (month) |  |  |
|  | Physicians rank | 4 | 1 | 5 | 3 | 6 | 2 |  |  |
|  | Patients rank | 2 | 1 | 6 | 4 | 5 | 3 |  |  |
| Bröckelmann, P. J. |  | 5Y-OS | 5Y-PFS | risk of SE requiring treatment | risk of peripheral neuropathy | risk of infertility | risk of permanent pulmonary toxicity |  |  |
|  | Physicians rank | 1 | 2 | 6 | 5 | 4 | 3 |  |  |
|  | Patients rank | 2 | 1 | 5 | 3 | 4 | 3 |  |  |
| Liu, F. X. |  | OS | ORR | PFS | risk of Ⅲ/Ⅳ SE | mode of administration | dosing schedule | MDT |  |
|  | Physicians rank | 2 | 3 | 4 | 1 | 6 | 5 | 6 |  |
|  | Patients rank | 1 | 3 | 4 | 2 | 7 | 5 | 6 |  |
| Landfeldt, E. |  | OS | PFS | degree of fatigue | degree of nausea | risk of serious infections | mode & frequency of administration |  |  |
|  | Physicians rank | 1 | 4 | 3 | 6 | 2 | 5 |  |  |
|  | Patients rank | 1 | 5 | 4 | 6 | 2 | 3 |  |  |
| Park, M. H. |  | PFS | risk of bone marrow suppression | risk of hand-foot skin reaction | risk of gastrointestinal perforation | risk of bleeding | mode of administration |  |  |
|  | Physicians rank | 4 | 1 | 2 | 3 | 3 | 3 |  |  |
|  | Patients rank | 1 | 3 | 5 | 6 | 4 | 2 |  |  |
| Thrumurthy, S. G. |  | mortality | morbidity | cure rate | QoL | hospital type | surgeon’s reputation |  |  |
|  | Physicians rank | 2 | 5 | 3 | 1 | 6 | 4 |  |  |
|  | Patients rank | 5 | 3 | 2 | 1 | 6 | 4 |  |  |
| Muhlbacher, A. C. |  | increase in life-span | further therapies | self-medication | breaks in treatment | emotional situation | physical situation |  |  |
|  | Physicians rank | 1 | 2 | 3 | 5 | 4 | 6 |  |  |
|  | Patients rank | 2 | 1 | 5 | 4 | 3 | 6 |  |  |
| DCE, Discrete Choice Experiment; CA, Conjoint Analysis; QoL, Quality of Life; SE, Side Effect; PFS, Progression-free Survival; 3Y-PL, Probability of Living at Least 3 Years; 5Y-OS, 5 Years Overall Survival; 5Y-PFS, 5 Years Progression-free Survival; MDT, Median Duration of Therapy; ORR, Objective Response Rate; OS, Overall Survival; Ⅲ/Ⅳ SE, Ⅲ/Ⅳ side effects | | | | | | | | | |
